# Constraints on the malaphoric $B_3-L_2$ model from di-lepton resonance searches at the LHC

**DOI:** 10.1140/epjc/s10052-025-14007-1

**Published:** 2025-03-14

**Authors:** Ben Allanach

**Affiliations:** DAMTP, University of Cambridge, Wilberforce Road, Cambridge, CB3 0WA UK

## Abstract

We confront the malaphoric $B_3-L_2$ model with bounds coming from a search for resonances in the di-lepton channels at the 13 TeV LHC. In contrast to the original $B_3-L_2$ model, the $Z^\prime$ of the *malaphoric* $B_3-L_2$ model has sizeable couplings to the lighter two families; these originate from order unity kinetic mixing with the hypercharge gauge boson and ameliorate the fit to lepton flavour universality measurements in *B*-meson decays. The $Z^\prime$ coupling to the first two families of quark means that the resulting constraints from resonant di-lepton searches are stronger. Nevertheless, we find that for $M_{Z^\prime }>2.8$ TeV there remains a non-negligible region of allowed parameter space where the model significantly improves upon several Standard Model predictions for observables involving the $b \rightarrow s l^+ l^-$ transition. We estimate that the 3000 fb$^{-1}$ HL-LHC will extend this sensitivity to $M_{Z^\prime }= 4.2$ TeV.

## Introduction

The malaphoric $B_3-L_2$ model was introduced in Ref. [1] in order to ameliorate fits of the Standard Model (SM) to current *B* meson decay observables involving the ${\bar{b}} s \mu ^+ \mu ^-$ and ${\bar{b}} s e^+ e^-$ effective couplings (and their CP conjugates^1^). There are significant theoretical uncertainties in the predictions of many of these observations, notably from the contribution from non-local hadronic matrix elements. The largest theory uncertainty, estimated from a combination of data and theory in Ref. [2], is from the charm loop contribution. There are concerns that even the state-of-the-art calculations do not completely capture all of the contributions; in particular loop diagrams involving effective hadrons running in the loop [3]. Although no estimate of all such contributions has been yet completed, recent estimates [2, 4, 5] have bounded various different contributions to it, finding that they are too small to explain the current disparity between measurements and state-of-the-art SM predictions. It is fair to say, however [3], that the SM predictions for such observables require further work before evidence for new physics can be unambiguously claimed. We shall investigate the case that the additional effective hadronic contributions are small and we shall fit a new physics contribution to the measurements.

One such new physics model that explains this new physics contribution is the malaphoric $B_3-L_2$ model. The malaphoric $B_3-L_2$ model is a deformation of the original $B_3-L_2$ model [6–8] to allow for sizeable kinetic mixing. In either $B_3-L_2$ model, an extra spontaneously broken $U(1)_X$ gauge group is introduced in a direct product with the SM gauge group. The *X* charges of the SM fermions are assumed to be third family baryon number minus second family lepton number, and the model requires the addition of at least one SM-singlet right-handed Weyl fermionic field (a right-handed neutrino) for anomaly cancellation. Right-handed neutrinos also provide a simple means of obtaining tiny neutrino masses via the seesaw mechanism and the model has a total of three. In contrast to the SM gauge group, the $B_3-L_2$ gauge group distinguishes between the different families. A SM-singlet complex scalar field $\theta$ (the ‘flavon’) that has a non-zero *X* charge $X_\theta$ is assumed to acquire a non-zero vacuum expectation value $\langle \theta \rangle \sim {{\mathcal {O}}(1)}$ TeV and spontaneously break $U(1)_X$. Assuming that the SM Higgs doublet field has a zero *X* charge leads to an attractive property of the model: all off-diagonal CKM matrix elements are predicted to be much smaller than unity aside from $|V_{us}|$ and $|V_{cd}|$, agreeing with measurements [9]. With the $U(1)_X$ gauge symmetry spontaneously broken, a massive spin-1 vector boson field $X^\mu$ remains, whose mass parameter

1 $$\begin{aligned} M_X= \frac{g_X}{\sqrt{2}} X_\theta \langle \theta \rangle \end{aligned}$$

is expected to be around the TeV scale. This electrically-neutral and colourless spin-1 bosonic field (the associated particle is dubbed a $Z^\prime$ boson) has tree-level flavour changing neutral currents. However, if one assumes that these are mostly associated with the transitions of the up quarks and of neutrinos, they are not subject to constraints on new physics coming from tree-level flavour changing currents between the *d* and *s* quark fields, which are extremely tight and derive from measurements of $K-{\bar{K}}$ mixing. In the years leading up to December 2022, new physics effects in the ${\bar{b}} s \mu ^+ \mu ^-$ effective coupling provided a much better fit to *B*-meson decay data than did the SM. In the $B_3-L_2$ model, this effective coupling is mediated at tree-level by the $Z^\prime$ boson as well as by loops involving electroweak gauge bosons. Since December 2022 though, analyses from the LHCb collaboration indicate that a better fit results when there is a new physics contribution to the ${\bar{b}} s e^+ e^-$ effective coupling in addition to that of ${\bar{b}} s \mu ^+ \mu ^-$ [10].

The *malaphoric* $B_3-L_2$ model achieves this by introducing a substantial kinetic mixing between the hypercharge gauge boson field $B^\mu$ and the $X^\mu$ gauge boson; such a term is invariant under all of the symmetries in the model. Since $B^\mu$ has interactions with both di-electron pairs and di-muon pairs, the kinetic mixing term results in the $Z^\prime$ boson also having these interactions. Ref. [1] demonstrates that this results in a significantly better global fit to $b\rightarrow s l^+ l^-$ transition measurements^2^, than does the original model. Ref. [1] used the language of deriving SMEFT coefficients from integrating out the $Z^\prime$. This was appropriate because all of the constraints applied were on measurements at energy scales much less than the mass of the $Z^\prime$, $M_{Z^\prime }$. Here, we shall need a different treatment because we will (in addition) apply constraints coming from LHC resonance searches. These necessarily involve observables measured at a scale of the $M_X$ and so the SMEFT cannot be used to characterise them.

We shall therefore introduce the malaphoric $B_3-L_2$ model in the effective field theory that *includes* the associated $Z^\prime$, rather than having it integrated out of the theory as in the SMEFT characterisation. Our discussion roughly follows (and agrees with) Refs. [11, 12], but has a more compact notation. We have three gauge fields which are electrically neutral and which mix. In the model gauge eigenbasis, these are put into a 3-vector (vectors will be written here in bold typeface) ${\hat{\textbf{G}}}_\mu :=({\hat{B}}_\mu ,\ {\hat{W}}^3_\mu ,\ {\hat{X}}_\mu )^T$, where the circumflex denotes that the field is in the original gauge kinetically-mixed basis and ${\hat{W}}^3_\mu$ is the electrically neutral *SU*(2) gauge field. We begin with the relevant important terms of the Lagrangian density involving ${\hat{\textbf{G}}}$:2where objects with two Lorentz indices indicate the field strength, *K* is a matrix encoding the kinetic mixing and $M^2_\mathbf{\hat{G}}$ is the neutral gauge boson mass squared matrix

3 $$\begin{aligned}  &   K:=\begin{pmatrix} 1 &  0 &  \sin \chi \\ 0 &  1 &  0 \\ \sin \chi &  0 &  1 \\ \end{pmatrix}, \qquad \nonumber \\  &   M^2_{\hat{\textbf{G}}}:= \begin{pmatrix} {g'}^2 v^2/4 &  -gg' v^2/ 4 &  0 \\ -gg' v^2/4 &  {\hat{g}}^2v^2/4 &  0 \\ 0 &  0 &  M_X^2 \\ \end{pmatrix}. \end{aligned}$$

$M^2_{\hat{\textbf{G}}}$ comes about as a consequence of the SM Higgs boson doublet field H acquiring a vacuum expectation value $\langle H \rangle = (0, v/\sqrt{2})^T$. We have parameterised the kinetic mixing terms by $\sin \chi$ in order to enforce that its magnitude is less than unity: otherwise, one of the eigenstates would have an incorrect kinetic term sign in the Lagrangian. We have also introduced the 3-vector

4 $$\begin{aligned} {\hat{\textbf{l}}}:= \left( g' {\hat{Y}},\ g {\hat{L}},\ g_X {\hat{X}}\right) ^T \end{aligned}$$

where $g'$ is the hypercharge gauge coupling, *g* the *SU*(2) gauge coupling and $g_X$ the $U(1)_X$ gauge coupling. The sum in (2) is over the left and right-handed Weyl fermionic gauge eigenstate representations of the model, $\psi ^\prime$ (Table 1).

**Table 1:** Representation of gauge eigenstates of Weyl fermion fields under the $SU(3)\times SU(2) \times U(1)_Y \times U(1)_X$ gauge symmetry of the $B_3-L_2$ model

Field $\psi$	$q_i^\prime =(u_{L_i}^\prime ,\ d_{L_i}^\prime )^T$	$l_i^\prime =(e_{L_I}^\prime ,\ \nu _{L_i}^\prime )$	$e_i^\prime$	$d_i^\prime$	$u_i^\prime$	$\nu _i^\prime$
*SU*(3)	3	1	1	3	3	1
*SU*(2)	2	2	1	1	1	1
$Y_\psi$	1/6	$-1/2$	$-1$	$-1/3$	2/3	0
$X_\psi$	$\delta _{i3}$	$-3\delta _{i2}$	$-3\delta _{i2}$	$\delta _{i3}$	$\delta _{i3}$	$-3\delta _{i2}$

In (4), the hat denotes an operator, thus ${\hat{Y}} \psi ^\prime = Y_{\psi ^\prime } \psi ^\prime$, ${\hat{X}} \psi ^\prime = X_{\psi ^\prime } \psi ^\prime$ and ${\hat{L}}$ annihilates (anti-)right-handed Weyl fermionic fields or returns an eigenvalue of $T_3/2$ on an *SU*(2) doublet field, where $T_3$ is the diagonal generator of the Lie algebra of *SU*(2) (one half of the third Pauli matrix in our conventions, in *SU*(2) fundamental index space).

The kinetic terms are diagonalised by the similarity transform $P_\chi ^{-1} K P_\chi$ with a non-unitary invertible matrix

5 $$\begin{aligned} P_\chi := \begin{pmatrix} 1 &  0 &  - \tan _\chi \\ 0 &  1 &  0 \\ 0 &  0 &  1/\cos _\chi \\ \end{pmatrix}. \end{aligned}$$

${\hat{M}}^2_{\hat{\textbf{G}}}$ is diagonalised by $O_w^T {\hat{M}}^2_{\hat{\textbf{G}}} O_w$ with the orthogonal matrix

6 $$\begin{aligned} O_{{\hat{w}}}:= \begin{pmatrix} {\hat{c}}_w &  -{\hat{s}}_w &  0 \\ {\hat{s}}_w &  {\hat{c}}_w &  0 \\ 0 &  0 &  1\\ \end{pmatrix}, \end{aligned}$$

yielding eigenvalues 0 (with the photon field $A_\mu$ as its eigenvector), $M_{{\hat{Z}}}^2:=v^2 (g^2+{g'}^2)/4$ (we call the corresponding eigenvector ${\hat{Z}}_\mu$), and $M_X^2$, whose eigenvector is $X_\mu$. Here, ${\hat{c}}_w:=\cos {\hat{\theta }}_w$ and ${\hat{s}}_w:=\sin {\hat{\theta }}_w$. ${\hat{\theta }}_w$ is the would-be weak mixing angle in the kinetically-mixed basis, distinct from the measured weak mixing angle $\theta _w$. The relationship between $\theta _w$ and ${\hat{\theta }}_w$ will be detailed below.

For brevity, we shall use the notation $t_\chi :=\tan \chi$, $c_\chi := \cos \chi$ and $s_\chi :=\sin \chi$. Writing the neutral gauge fields in a new basis $\textbf{H}_\mu :=O_{{\hat{w}}}^T P_\chi ^{-1} {\hat{\textbf{G}}}_\mu$, (2) becomes7where

8 $$\begin{aligned} M_\textbf{H}^2:= \begin{pmatrix} 0 &  0 &  0 \\ 0 &  M_{{\hat{Z}}}^2 &  M_{{\hat{Z}}}^2 {\hat{s}}_w t_\chi \\ 0 &  M_{{\hat{Z}}}^2 {\hat{s}}_w t_\chi &  M_X^2 / c_\chi ^2 + M_X^2 {\hat{s}}_w^2 t_\chi ^2 \\ \end{pmatrix} \end{aligned}$$

which is diagonalised by a similarity transform $O_z^T M_\textbf{H}^2 O_z$ with the orthogonal matrix

9 $$\begin{aligned} O_z:= \begin{pmatrix} 1 &  0 &  0 \\ 0 &  c_z &  -s_z \\ 0 &  s_z &  c_s \\ \end{pmatrix} \end{aligned}$$

provided that

10 $$\begin{aligned} \tan 2 \theta _z = \frac{-2 M_{{\hat{Z}}}^2 {\hat{s}}_w s_\chi c_\chi }{M_X^2 + M_{{\hat{Z}}}^2 ({\hat{s}}_w^2 s_\chi ^2 - c_\chi ^2)}. \end{aligned}$$

We define the propagating (or ‘physical’) eigenstates $\textbf{P}_\mu :=(A_\mu ,\ Z_\mu ,\ Z^\prime _\mu )^T:= O_z^T O_{{\hat{w}}}^T P_\chi ^{-1} {\hat{\textbf{G}}}_\mu$ to write11where

12 $$\begin{aligned} C:=O_{{\hat{w}}}^T P_\chi O_{{\hat{w}}} O_z \end{aligned}$$

and $M_\textbf{P}^2=\text {diag}(0, M_Z^2, M_{Z^\prime }^2)$, where $M_Z$ and $M_{Z^\prime }$ are the physical $Z^0$ and $Z^\prime$ boson masses, respectively.

The physical parameters are related to the model’s parameters by [12]

13 $$\begin{aligned} M_{{\hat{Z}}}^2= &   M_Z^2 \left[ 1 + s_z^2 \left( \frac{M_{Z^\prime }^2}{M_Z^2}-1\right) \right] , \end{aligned}$$

14 $$\begin{aligned} M_{X}^2= &   \frac{c_\chi ^2}{1+{\hat{s}}_w^2 s_\chi ^2} \left[ s_z^2 M_Z^2 + c_z^2 M_{Z^\prime }^2 \right] , \end{aligned}$$

15 $$\begin{aligned} M_{{\hat{Z}}} {\hat{c}}_w {\hat{s}}_w= &   M_Z c_w s_w \end{aligned}$$

along with (10). $c_w$ is the cosine of the weak mixing angle measured by experiments; $c_w = M_W/M_Z$ where $M_W$ is the *W*-boson mass. We wish to solve the system of equations (10), (13)–(15) for a given $M_Z$ (taken from experimental measurement), $M_{Z^\prime }$ and $s_\chi$. We shall solve these equations using approximation which is good enough for our purposes here in Sect. 2 and detail an iterative procedure in order to obtain an arbitrarily close approximation numerically in Appendix A.

The final term in (11) yields the coupling of the $Z^\prime$ to the gauge eigenstates of the fermions $\psi ^\prime$. In order to make contact with phenomenology, we need to know the couplings of the $Z^\prime$ to the mass eigenstates of the fermionic fields

16 $$\begin{aligned} \psi _i:= (V_{\psi }^\dag )_{ij} \psi ^\prime _j, \end{aligned}$$

where $V_{\psi }$ is a 3 by 3 unitary matrix in family space and $i,j,\in \{1,2,3\}$ are family indices (with an implicit Einstein summation convention). Following Ref. [1], we assume that, to a good approximation, $V_{l_L}=V_{e_R}=V_{u_R}=V_{d_R}=I_3$, the 3 by 3 identity matrix, whereas

17 $$\begin{aligned} V_{d_L}:= \begin{pmatrix} 1 &  0 &  0 \\ 0 &  c_{sb} &  s_{sb} \\ 0 &  -s_{sb} &  c_{sb} \\ \end{pmatrix}. \end{aligned}$$

$\theta _{sb}\ne 0$ will then facilitate tree-level $b \rightarrow s$ transitions via $Z^\prime$ couplings. We then obtain $V_{u_L}=V_{CKM}^\dag V_{d_L}$ and $V_{\nu _L}=V_{PMNS}^\dag$, where $V_{CKM}$ is the Cabbibo-Kobayashi-Maskawa matrix and $V_{PMNS}$ is the Pontecorvo–Maki–Nakagawa–Sakata matrix [9]. The assumptions above about the $V_\psi$ are strong, but have the consequence that strong constraints from some flavour changing $Z^\prime$ couplings are evaded (for example from charged lepton flavour violating processes).

The final term in (11) can be expanded in terms of the mass eigenstates in order to calculate the $Z^\prime$ couplings in the malaphoric $B_3-L_2$ model, on which the phenomenology of the model heavily depends. We shall perform this expansion in Sect. 2. In particular, a family universal part coming from the kinetic mixing will provide $Z^\prime$ couplings to the first two families of quark, enhancing the LHC production cross section. We shall then analytically solve (10), (13)–(15) approximately. In Sect. 3, we detail how we use the ATLAS di-lepton searches to place bounds upon the parameter space of the model. The results of this are then shown before concluding in Sect. 4.

## $Z^\prime$-fermion couplings in the malaphoric $B_3-L_2$ model

From now on, we shall write a 3-vectors of fermion fields in family space in bold: ${\varvec{\psi }}:=(\psi _1,\ \psi _2,\ \psi _3)^T$. Expanding the right-most term of (11) in terms of the mass eigenstates of these, we obtain the coupling of the $Z^\prime$ bosonic field to them18where $\Lambda _\xi ^{\psi }:=V_\psi ^\dag \xi V_\psi$,

19 $$\begin{aligned} \xi := \begin{pmatrix} 0 &  0 &  0 \\ 0 &  0 &  0 \\ 0 &  0 &  1 \\ \end{pmatrix}, \qquad \Xi := \begin{pmatrix} 0 &  0 &  0 \\ 0 &  1 &  0 \\ 0 &  0 &  0 \\ \end{pmatrix}, \end{aligned}$$

and the $g^\psi$ are the family universal components of the $Z^\prime$ coupling to fermions:

20 $$\begin{aligned} g^{u_L}:= &   {\hat{g}}_Z \left( \frac{1}{2} - \frac{2}{3} {\hat{s}}_w^2 \right) \left( c_z {\hat{s}}_w t_\chi - s_z \right) - \frac{2}{3} e c_z {\hat{c}}_w t_\chi , \nonumber \\ g^{d_L}:= &   {\hat{g}}_Z \left( -\frac{1}{2} + \frac{1}{3} {\hat{s}}_w^2 \right) \left( c_z {\hat{s}}_w t_\chi - s_z \right) + \frac{e}{3} c_z {\hat{c}}_w t_\chi , \nonumber \\ g^{u_R}:= &   {\hat{g}}_Z \left( - \frac{2}{3} {\hat{s}}_w^2 \right) \left( c_z {\hat{s}}_w t_\chi - s_z \right) - \frac{2}{3} e c_z {\hat{c}}_w t_\chi , \nonumber \\ g^{d_R}:= &   {\hat{g}}_Z \frac{{\hat{s}}_w^2}{3} \left( c_z {\hat{s}}_w t_\chi - s_z \right) + \frac{e}{3} c_z {\hat{c}}_w t_\chi , \nonumber \\ g^{\nu _L}:= &   \frac{{\hat{g}}_Z}{2} \left( c_z {\hat{s}}_w t_\chi - s_z \right) , \nonumber \\ g^{e_L}:= &   {\hat{g}}_Z \left( -\frac{1}{2} + {\hat{s}}_w^2 \right) \left( c_z {\hat{s}}_w t_\chi - s_z \right) - e c_z {\hat{c}}_w t_\chi , \nonumber \\ g^{e_R}:= &   {\hat{g}}_Z {\hat{s}}_w^2 \left( c_z {\hat{s}}_w t_\chi - s_z \right) + e c_z {\hat{c}}_w t_\chi , \end{aligned}$$

where ${\hat{g}}_Z:= e / ({\hat{s}}_w {\hat{c}}_w)$ and *e* is the electromagnetic gauge coupling.

As we mention in §1, the malaphoric $B_3-L_2$ was fit via the SMEFT to various measurements in Ref. [1]: $b \rightarrow s$ transition observables and di-lepton scattering cross sections.^3^ The SMEFT was expanded in powers of $g_X / M_X$ and the 95$\%$ CL region of good fit was phrased in terms of $\{g_X/M_X,\ s_\chi /M_X,\ \theta _{sb}\}$. In a total of 509 observables, the $\chi ^2$ was found to improve upon the SM value by 40.1 units for a best-fit point

21 $$\begin{aligned}  &   g_X\frac{\text {3~TeV}}{M_X} = 0.048,\qquad \sin \chi \frac{\text {3~TeV}}{M_X} = -0.86,\qquad \nonumber \\  &   \theta _{sb}=-0.19. \end{aligned}$$

The di-lepton bump hunt at the LHC is phrased in terms of $M_{Z^\prime }$ however, and we must solve (10) and (13)–(15) for it. $M_Z$ has been measured with high precision and so we take it as an input here (we shall fix it to its experimental central value), as we do $s_w$. The final list of input parameters is then $\{ M_Z,\ s_w,\ s_\chi ,\ M_X\}$ whereas we need to solve the equations for $\{ \theta _z,\ {\hat{s}}_w,\ M_{{\hat{Z}}},\ M_{Z^\prime }\}$. We know of no exact analytic solution in closed form.

We shall make use of the fit region derived in Ref. [1] from the SMEFT given by integrating out the $Z^\prime$. Since the fit region used, among other observables at lower scales, cross-section measurements from LEP2 at a centre of mass energy of up to 206.5 GeV encoded via the SMEFT, SMEFT applicability requires that $M_{Z^\prime }$ should be much larger than this energy scale. This implies that we may use $\epsilon :=(M_Z/M_{Z^\prime })^2\ll 1$ as a perturbative expansion parameter in order to find an approximate solution. Here, an approximate solution, valid up to a factor $(1+\mathcal {O}(\epsilon ))$, is easily precise enough for the purpose of predicting LHC $Z^\prime$ production cross sections, which have uncertainties larger than the order one percent (or less) uncertainty coming from the perturbation expansion:

22 $$\begin{aligned} \theta _z= &   - \frac{M_Z^2}{M_{Z^\prime }^2} s_w t_\chi (1 + s_w^2 s_\chi ^2), \end{aligned}$$

23 $$\begin{aligned} {\hat{s}}_w= &   s_w, \end{aligned}$$

24 $$\begin{aligned} M_{{\hat{Z}}}= &   M_Z, \end{aligned}$$

25 $$\begin{aligned} M_{Z^\prime }= &   \frac{\sqrt{1+ s_w^2 s_\chi ^2}}{c_\chi } M_{X}. \end{aligned}$$

We remark that (22)–(25) provide a valid approximation for any $-1< s_\chi < 1$ and we note that $Z-Z^\prime$ mixing is small: $\theta _z \sim {\mathcal {O}}(\epsilon )$. The malaphoric $B_3-L_2$ model described here has been encoded (neglecting the flavon field $\theta$) in UFO [13] format using (22)–(25) and made available in the ancillary directory of the arXiv preprint of this paper.

(10) and (13)–(15) could instead be solved numerically to arbitrary precision by a fixed point iteration method, as we detail in Appendix A. A more precise method such as the one in Appendix A could be useful in the future if (for example) one wished to use very precise experimental data from a future high energy $e^+ e^-$ collider such as the FCC-ee [14] or CEPC [15] to bound the model.

## LHC $Z^\prime$ production cross-section and fit region

The re-casting of a 139 fb$^{-1}$ 13 TeV ATLAS search [16] for *pp* to resonant di-lepton production follows that of Ref. [17] but we shall describe it in brief here. ATLAS observed no significant excess on SM backgrounds and provides an observed 95$\%$ upper bound on $s(z, M_{Z^\prime })$, the total production cross-section multiplied by branching ratio of a new physics state decaying to a specified flavour of di-lepton pair (either di-electrons or di-muons). Here, *z* is defined to be the new physics state’s resonance width divided by its mass. ATLAS provides the bounds upon both a narrow width $s(0, M_{Z^\prime })$ and a resonance whose width is a tenth of its mass, $s(0.1, M_{Z^\prime })$. The function

26 $$\begin{aligned} s(z, M_{Z^\prime }) = s(0, M_{Z^\prime }) \left( \frac{s(0.1, M_{Z^\prime })}{s(0,M_{Z^\prime })} \right) ^{z/10} \end{aligned}$$

has been demonstrated to provide a good approximation for some other values of *z* between 0 and 0.1 that were also used by ATLAS for the di-electron channel [17] as well as for the di-muon channel [18]. We shall use a superscript on *s* to denote the lepton flavour, i.e. $s^{\mu ^+\mu ^-}$ or $s^{e^+e^-}$. Here, we shall use (26) to interpolate for $0\le z \le 1$ and to extrapolate for $z > 0.1$, although if there is a region of parameter space on a plot that has extrapolation, we shall demarcate it the plot in question, since validation of (26) has not been performed there.

As mentioned above, the malaphoric $B_3-L_2$ model was fit via the SMEFT to LEP2 data, to $b \rightarrow s$ transition data and electroweak precision observables in Ref. [17]. The fit depends, to a good approximation, on only three effective beyond-the-SM parameters which can be taken to be $(\sin \chi /M_X)$, $(g_X/M_X)$ and $\theta _{sb}$. On the other hand, the collider physics depends sensitively on $\sin \chi$, $M_X$ and $g_X$ but in practice, not sensitively on $\theta _{sb}$. In the parameter region considered, $\sin \theta _{sb}$ is not close to unity [1], where the $s {\bar{s}} \rightarrow Z^\prime$ channel could change appreciably and alter the production cross-section.

**Table 2:** Example 13 TeV LHC $Z^\prime$ production cross-section contributions and other quantities of interest for $M_X=4$ TeV, $s_\chi =-0.2$, $g_X=0.1$. We have neglected to list any cross-section contributions below the $10^{-5}$ fb level

	$\theta _{sb}=-0.19$	$\theta _{sb}=0$
$M_{Z^\prime }$/TeV	4.184	4.184
$\Gamma _{Z^\prime }$/GeV	30.5	30.5
$BR(Z^\prime \rightarrow e^+ e^-)$	0.01	0.01
$BR(Z^\prime \rightarrow \mu ^+ \mu ^-)$	0.48	0.48
$BR(Z^\prime \rightarrow t {\bar{t}})$	0.20	0.21
$BR(Z^\prime \rightarrow b {\bar{b}})$	0.08	0.08
$\sigma (u {\bar{u}} \rightarrow Z^\prime \rightarrow \mu ^+ \mu ^)$/fb	0.034	0.034
$\sigma (d {\bar{d}} \rightarrow Z^\prime \rightarrow \mu ^+ \mu ^)$/fb	0.0032	0.0032
$\sigma (c {\bar{c}} \rightarrow Z^\prime \rightarrow \mu ^+ \mu ^)$/fb	3.2 $\times 10^{-4}$	3.1 $\times 10^{-4}$
$\sigma (s {\bar{s}} \rightarrow Z^\prime \rightarrow \mu ^+ \mu ^)$/fb	1.4 $\times 10^{-4}$	1.1 $\times 10^{-4}$
$\sigma (b {\bar{b}} \rightarrow Z^\prime \rightarrow \mu ^+ \mu ^)$/fb	6.2 $\times 10^{-4}$	6.5 $\times 10^{-4}$
$\sigma (p p \rightarrow Z^\prime b \rightarrow \mu ^+ \mu ^- b)$/fb	3.6 $\times 10^{-5}$	3.4 $\times 10^{-5}$
$\sigma (p p \rightarrow Z^\prime \rightarrow e^+ e^-)$/fb	9.1 $\times 10^{-4}$	9.1 $\times 10^{-4}$
$s^{\mu ^+\mu ^-}$	0.048	0.048
$s^{e^+ e^-}$	0.037	0.037

We estimate the LHC production cross-section and $Z^\prime$ total width and decay rates using MadGraph2.9.21 at tree-level order [19]. We pick an example point in parameter space from Ref. [17] which is just within the 95$\%$ fit window and show various quantities of interest in Table 2. The non-zero value of $\theta _{sb}$ chosen is taken from its best-fit value, given the other parameters chosen. From the table, we see by comparing the numerical values of the two rightmost columns that changing $\theta _{sb}$ does not change the branching ratios or $Z^\prime$ production cross-section times branching ratio much. This qualitative statement holds over the rest of the parameter space that we shall cover. From now on in the present paper, in our estimates for the cross-section, we shall approximate $\theta _{sb}=0$ when calculating the limits coming from LHC resonant di-lepton production.

We see from the table that the partonic channel dominating the $Z^\prime$ production cross-section is via $u {\bar{u}}\rightarrow Z^\prime$: this proceeds via the hypercharge mixing and is an order of magnitude larger than the $d {\bar{d}}$ contribution because of a combination of parton distribution functions and the larger hypercharge of $u_R$ quarks (implying a larger $Z^\prime$ coupling to them via the kinetic mixing mixing). We also see that the associated production $pp \rightarrow Z^\prime b$ is small. This associated production cross-section can be of a similar order to that of direct $p p \rightarrow Z^\prime$ in the case where the $b {\bar{b}}$ production channel dominates, as it did in the original, kinetically unmixed $B_3-L_2$ model. In that case, a CMS analysis [20] searching for resonant di-muons plus *b*-jets can be used to acquire extra sensitivity. Here though, it should only be relevant near the $s_\chi \rightarrow 0$ limit, which (as we shall see below) is outside the region of good fit to flavour and other data. We shall therefore concentrate on the inclusive resonant di-lepton production cross-sections.

**Fig. 1:**
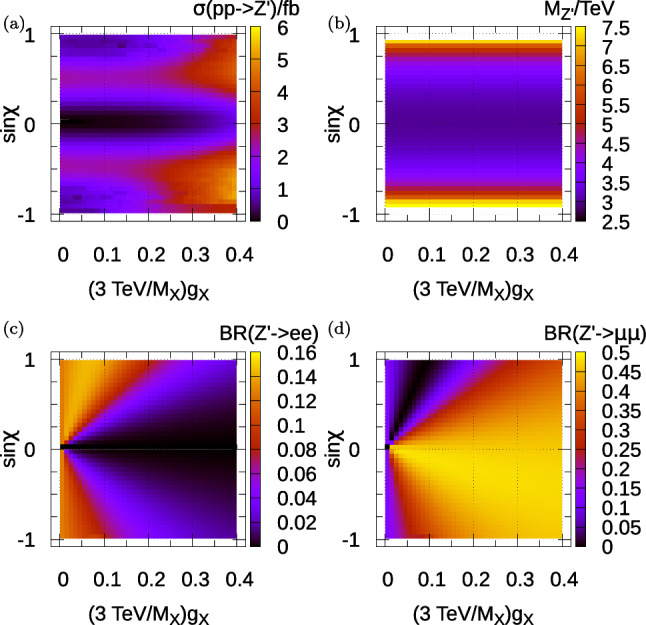
Properties of the parameter space of the malaphoric $B_3-L_2$ model at $M_X=3$ TeV. **a** Displays the $Z^\prime$ production cross-section at the 13 TeV LHC. **b**–**d** Show various $Z^\prime$ properties as labelled on the colour bar. $\sigma (pp\rightarrow Z^\prime )$ is not sensitive to the value of $\theta _{sb}$ and $\theta _{sb}=0$ has been taken in its calculation

To begin with, we fix $M_X=3$ TeV and vary $\sin \chi$ and $g_X/M_X$ (i.e. effectively varying $g_X$). We have picked the domain of the abscissa to encompass the region of good fit, as we shall see in Fig. 3. The 13 TeV LHC $Z^\prime$ total production cross section is shown in Fig. 1a. The $Z^\prime$ total production cross section increases towards the right-hand side of the plot, where the gauge coupling is larger. It becomes small when $s_\chi \rightarrow 0$ because there the $u {\bar{u}}$ coupling to the $Z^\prime$ vanishes. $M_{Z^\prime }$ varies across this two-parameter plane according to (25) and is shown in Fig. 1b. We see that it is around 3 TeV except towards $|\sin \chi |=1$, where $M_{Z^\prime }$ reaches up to 7 TeV. Near $s_\chi =1$, the total cross-section is suppressed because of the effects of this large mass, as a quick reference to Fig. 1a confirms. The two relevant leptonic branching ratios $BR(Z^\prime \rightarrow e^+ e^-)$ and $BR(Z^\prime \rightarrow \mu ^+ \mu ^-)$ are shown in Fig. 1c, d, respectively. Note that, to a good approximation, this model predicts that $BR(Z^\prime \rightarrow \tau \tau )=BR(Z^\prime \rightarrow ee)$.

**Fig. 2:**
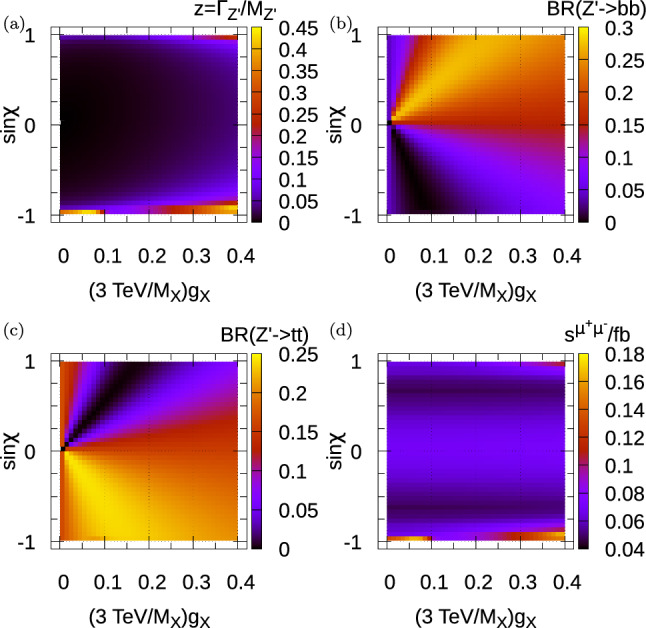
$Z^\prime$ properties of the malaphoric $B_3-L_2$ model at $M_X=3$ TeV. **a** Shows the width of the $Z^\prime$ divided by its mass. **b** Shows the branching ratio of the $Z^\prime$ into $b {\bar{b}}$ pairs, **c** Shows the branching ratio into $t {\bar{t}}$ pairs and **d** Shows the observed 95$\%$ CL upper bound on total $pp\rightarrow Z^\prime \rightarrow \mu ^+\mu ^-$ cross-section derived from the 13 TeV ATLAS 139 fb$^{-1}$ resonant di-lepton search [16]. $\sigma (pp\rightarrow Z^\prime )$ is not sensitive to the value of $\theta _{sb}$ and $\theta _{sb}=0$ has been taken in its calculation

Two other branching ratios of the $Z^\prime$ (into $b {\bar{b}}$ and $t {\bar{t}}$ final states) and the total width of the $Z^\prime$ boson divided by its mass are shown in Fig. 2. The upper bounds on resonant $Z^\prime$ production followed by decay into $\mu ^+\mu ^-$ coming from the resonant ATLAS di-lepton search over this parameter space are functions of the production cross-section, the relevant leptonic branching ratio as well as *z* and $M_{Z^\prime }$. The fraction of $Z^\prime$ width over its mass is displayed in the figure. The fact that it is less than 0.45 means that nowhere in parameter space is the resonance frequency becoming so wide that perturbation theory is obviously breaking down (this happens near fractions of around 1).

**Fig. 3:**
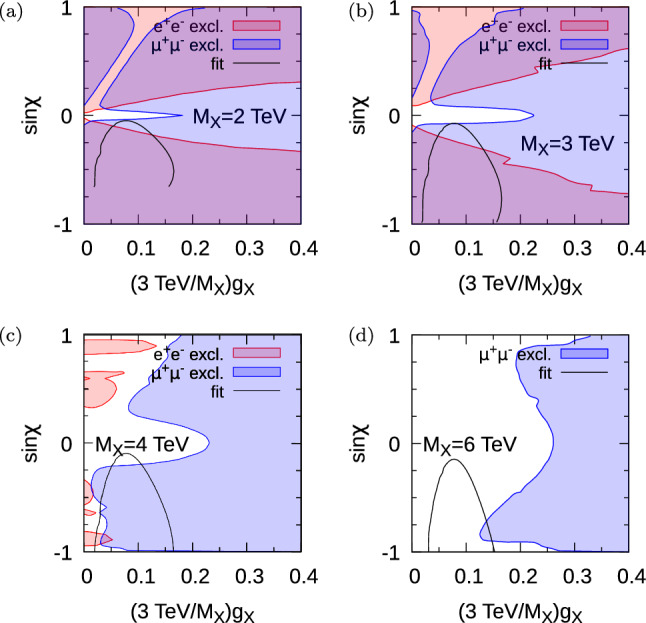
Parameter space of the malaphoric $B_3-L_2$ model at various values of $M_X$. Regions that are excluded at 95$\%$ CL [16] are shown by the colour in the legend. The region bounded within each black contour is preferred by fits to data including $b \rightarrow s l^+l^-$ at the 95$\%$ CL, from Ref. [1]. $\sigma (pp\rightarrow Z^\prime )$ is not sensitive to the value of $\theta _{sb}$ and $\theta _{sb}=0$ has been taken in its calculation

We now turn to constraints upon the parameter space. We show these for various different chosen values of $M_X$ in Fig. 3. The white region on each plot is currently allowed at the 95$\%$ CL by the ATLAS resonant di-lepton search [16]. The region within the black curve is preferred by the global fit to measurements of $b \rightarrow s$ observables, electroweak parameters and LEP2 di-lepton production cross-sections [1]. The locus of this curve depends upon $M_X$: to a good approximation (neglecting small renormalisation group effects between $M_X$ and $M_{Z}$) it depends only on the ratio $\sin \chi /M_X$ and the abscissa. In Ref. [1], the curve was determined for $M_X=3$ TeV for values of $\sin \chi \le 1$. For $M_X=2$ TeV, this translates into a curve at values of $\sin \chi \le 2/3$, explaining why the curve in Fig. 3a is incomplete. From Fig. 3a, b, we see that there the region of good fit is ruled out by the ATLAS resonant di-lepton search (in particular in the di-muon channel) for $M_X=2$ TeV and $M_x=3$ TeV, respectively. However, Fig. 3c, d show that $M_X=4$ TeV and $M_X=6$ TeV have some allowed parameter space in the region of good fit.

**Fig. 4:**
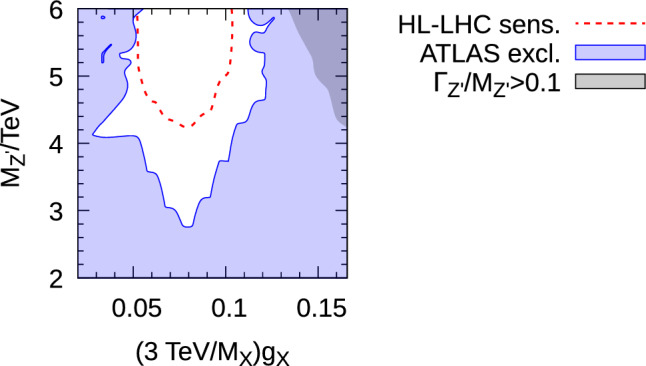
ATLAS di-lepton resonance search exclusions in terms of the physical mass of the $Z^\prime$ boson $M_{Z^\prime }$ [16]; the coloured region is excluded at the 95$\%$ CL whereas the dashed line shows the estimated HL-LHC sensitivity. In each case $\sin \chi$ has been adjusted to the value that gives the weakest constraint within the 95$\%$ fit region shown in Fig. 3 (see the text for more detail). $\sigma (pp\rightarrow Z^\prime )$ is not sensitive to the value of $\theta _{sb}$ and $\theta _{sb}=0$ has been taken in its calculation. The domain of the abscissa corresponds to the 95$\%$ CL region of good global fit from Ref. [1] when other parameters have been profiled over

Ideally, we wish to phrase the result of ATLAS searches in terms of the more physical parameter $M_{Z^\prime }$. We do this by using a 3-dimensional scan over $M_X$, $g_X$ and $\sin \chi$ within the 95$\%$ CL fit region. To effect this, we scan over the black curve in Fig. 3b parameterised jointly by the one-dimensional curve in $x:=(\text {3~TeV}/M_X)g_X$ and $y:=(\text {3~TeV}/M_X)\sin \chi$. $M_X$ is scanned between 2 and 6 TeV. $\sin \chi$ is then scanned between the value consistent with *y* and^4^$-0.95$ in steps of approximately^5^ 0.01. For each of these values of $\sin \chi$, we compute

27 $$\begin{aligned}  &   R{:=}\text {max}\left\{ \sigma (pp \rightarrow Z^\prime \rightarrow \mu ^+ \mu ^-)/s^{\mu ^+\mu ^-},\right. \nonumber \\  &   \left. \sigma (pp \rightarrow Z^\prime \rightarrow e^+ e^-)/s^{e^+e^-} \right\} . \end{aligned}$$

In the plot, $\sin \chi$ is then fixed to the scanned value that returns the minimum value of *R*. In this way, the approximate value of $\sin \chi$ is found which is the least excluded by the ATLAS di-lepton searches. If we then plot the contour $R=1$ across the $M_{Z^\prime }$ versus $(g_X/M_X)$ plane, we get a picture of the minimum amount of parameter space excluded (in other words, if we were to move $\sin \chi$ away from this value, we could exclude more parameter space). We plot the resulting exclusion in Fig. 4. We see that, within the 95$\%$ fit régime, one can infer that $M_{Z^\prime }>2.8$ TeV from the Run II ATLAS resonant di-lepton search whichever the value of the other parameters. Next, we wish to provide an estimate of the HL-LHC sensitivity to the model. For this, we re-purpose (27), first changing $s^{\mu ^+ \mu ^-}$ and $s^{e^+e^-}$ to the *expected bound* rather than the observed one. Since this may change the value of $\sin \chi$ which we use in the plot at each point (remembering that this is fixed to the value that returns the minimum value of *R*), the expected bound requires its own scan, which we perform. We then scale the expected ATLAS sensitivity by the square root^6^ of the luminosity. We assume that the HL-LHC will acquire 3000 fb$^{-1}$ of integrated luminosity and so the expected excluded cross-section is

28 $$\begin{aligned} s^\text {HL-LHC}=s (z, M_{Z^\prime }) \sqrt{\frac{139~\text {fb}^{-1}}{3000 \text {~fb}^{-1}}}. \end{aligned}$$

We see from Fig. 4 that our estimate yields that the HL-LHC should be sensitive up to at least $M_{Z^\prime }=4.2$ TeV at 95$\%$ CL.

## Conclusions

The original $B_3-L_2$ model explained some $b \rightarrow s \mu ^+ \mu ^-$ measurements,^7^ which were (prior to December 2022, unambiguously) in tension with SM predictions. It predicts a TeV-scale neutral $Z^\prime$ vector boson, which decays into di-muon pairs and could potentially be produced and found at the LHC. Direct search bounds were rather weak ($M_{Z^\prime }>1.2$ TeV in the region of good fit [23]) due to the fact that, to a good approximation, the LHC production cross-section was dominated by $b \bar{b} \rightarrow Z^\prime$ and so doubly suppressed by the *b*-quark parton distribution function. Since then, the measurements of lepton flavour universality (LFU) ratios $R_K$ and $R_{K^*}$ have changed, requiring a change to the model such that new physics effects in $b \rightarrow s e^+ e^-$ are also present. Here, we have taken the suggestion that the original model has sizeable kinetic mixing [1], and re-calculated LHC direct search bounds on the $Z^\prime$. In the new model, the $Z^\prime$ is dominantly produced at the LHC by valence quark production, with a consequently much higher cross-section. The bounds coming from direct searches at the LHC are then expected to be stronger than that of the original model. This is borne out by our analysis: ATLAS di-lepton searches at the 13 TeV LHC imply that the $Z^\prime$ boson of the malaphoric $B_3-L_2$ model should have a mass of at least 2.8 TeV if it is to explain the disparity between various current measurements of $b \rightarrow s$ transitions in *B*-meson decays and their state-of-the-art SM predictions while remaining compatible with measurements of electroweak observables and LEP2 di-lepton scattering measurements. The CMS experiment has also performed a similar di-lepton search [24], with a similar exclusion on cross-section multiplied by branching ratio to the ATLAS analysis used in the present paper as a function of $M_{Z^\prime }$. We expect bounds extracted from the CMS analysis to be very similar to those presented here.

In terms of direct signatures, we have ignored processes involving the flavon $\theta$. There is the possibility of a term in the potential $\lambda \theta ^\dag \theta H^\dag H$, which if $\lambda \ne 0$ leads to Higgs-flavon mixing. This would change Higgs decay and production rates [25], as well as leaving open the possibility of flavonstrahlung [8] via $p p \rightarrow Z^\prime \theta$. $\lambda$ is multiplicatively renormalised and therefore the $\lambda =0$ limit is stable to renormalisation corrections, so assuming that it is negligible is at least self-consistent. $\lambda \ne 0$ in the unmixed $B_3-L_2$ model has been studied in Ref. [25]. We leave studies of the phenomenology of sizeable values of $\lambda$ in the kinetically-mixed case to future work.

We note that a simple tweak to the malaphoric $B_3-L_2$ model leads to another possibility: one simply switches the *X* charges of the third and second family leptons in the model. The $Z^\prime$ coupling to the di-muon pairs would be entirely through the kinetic mixing terms. The di-electron pair coupling to $Z^\prime$ would be identical to the di-muon pair coupling, with the implication that lepton flavour should be universal between electrons and muons. The LFU observables taken as a whole still prefer a disparity between the di-electron and di-muon channels at the 2$\sigma$ level [26] and so this option is currently somewhat disfavoured. Another model possibility is that $X:=3B_3-L_1-2L_2$, shown in Ref. [27] to fit flavour, electroweak and LEP2 measurements well. In that case, there are no sizeable couplings to valence quarks and the current LHC bounds ($M_{Z^\prime } > 1.2$ TeV) are consequently weaker than those found in the present paper [17].

One may wonder about the possibilities for ultra-violet model building in the case with order unity kinetic mixing. We point out that the malaphoric model is equivalent to a model *with zero* kinetic mixing in which the charge is instead assigned to be $X:=B_3-L_2 + \alpha Y$, where $\alpha \in \mathbb {Q}$ is assigned appropriately (such charge assignments have been proposed in the literature [28, 29]).

In the case that $M_{Z^\prime }>4.2$ TeV, one would require future colliders to further probe the malaphoric $B_3-L_2$ model by resonant di-lepton searches, for example muon colliders or FCC-hh [30, 31]. The FCC-ee would provide diverse indirect evidence via flavour tagging [32], which would help test the model.

## Data Availability

My manuscript has no associated data. [Authors’ comment: Data sharing not applicable to this article as no datasets were generated or analysed during the current study.]

## A Iterative solution of kinetic mixing parameter equations

Here, we describe a method for the numerical solution of (10), (13)–(15) to arbitrary precision by fixed point iteration. We begin with an approximation for $M_{Z^\prime }$, $M_{{\hat{Z}}}$ and ${\hat{s}}_w$ and use the equations to derive a closer approximation. We shall use a suffix *i* on each of these quantities to denote the $i^{th}$ iteration. The initial iteration ($i=0$) is fixed by the right-hand sides of our approximate solution (22)–(25). First, we calculate

29 $$\begin{aligned} \theta _{z_i} = \frac{1}{2} \tan ^{-1} \left( \frac{-2 c_\chi s_\chi {\hat{s}}_{w_i} M^2_{{\hat{Z}}_i}}{M_{X}^2 + {M^2_{{{\hat{Z}}}_i}} ({\hat{s}}_{w_i}^2 s_\chi ^2 - c_\chi ^2)} \right) . \end{aligned}$$

This allows us to calculate the next iteration via

30 $$\begin{aligned} M_{{{\hat{Z}}}_{i+1}} = M_Z \sqrt{1 + s_{z_i}^2 \left( \frac{M_{Z^\prime _i}^2}{M_Z^2} - 1\right) }, \end{aligned}$$

which is substituted into

31 $$\begin{aligned} {\hat{s}}_{{2w_{i+1}}}= &   \frac{s_{2w} M_Z}{M_{{{\hat{Z}}}_{i+1}}}, \nonumber \\ M_{Z^\prime _{i+1}}= &   \frac{1}{c_{z_i} c_\chi } \sqrt{M_X^2(1 + {\hat{s}}_{w_{i+1}}^2 s_\chi ^2) - c_\chi ^2 s_{z_i}^2 M_Z^2} \end{aligned}$$

We find that this iterative procedure converges quickly: an example point ($M_{Z^\prime }=1$ TeV, $s_\chi =-0.86$) takes eight iterations to reach a relative precision of order $10^{-16}$. The algorithm and example case is provided in the ancillary directory associated with the arXiv version of this paper in the form of a python program.

## Footnotes

1 Throughout the rest of this article, CP conjugates are to be understood as to be added implicitly to the effective vertices we mention.

2 In the present paper, $l^\pm \in \{e^\pm ,\ \mu ^\pm \}$.

3 Throughout the rest of the article, this is the ‘fit’ referred to.

4 The parameter region $-1< \sin \chi < - 0.95$ leads to values of $M_{Z^\prime }>6$ TeV, i.e. higher than those relevant to the ATLAS bounds. As Fig. 2a shows, this region of parameter space also has wide $Z^\prime$ bosons, resulting from larger couplings, with less reliable perturbativity.

5 We scan $\sin \chi$ between $-0.95$ and the edge value $E=y M_X/\text {(3~TeV)}$ with $100(E-0.95)$ equally spaced steps, rounded down to the nearest integer.

6 For some potential caveats associated with such a procedure, see Ref. [21].

7 $B_i-L_j$ modes have also been used to explain measurements of the anomalous magnetic moment of the muon and thermal dark matter [22].

## Code Availability Statement

My manuscript has no associated code/software. [Authors’ comment: Code/Software sharing not applicable to this article as no code/software was generated or analysed during the current study.]
